# Insights into nanoparticles-induced neurotoxicity and cope up strategies

**DOI:** 10.3389/fnins.2023.1127460

**Published:** 2023-05-04

**Authors:** Sana Zia, Amjad Islam Aqib, Afshan Muneer, Mahreen Fatima, Khazeena Atta, Tasleem Kausar, C-Neen Fatima Zaheer, Irfan Ahmad, Mohd Saeed, Asyia Shafique

**Affiliations:** ^1^Department of Zoology, Government Sadiq College Women University, Bahawalpur, Pakistan; ^2^Department of Medicine, Cholistan University of Veterinary and Animal Sciences, Bahawalpur, Pakistan; ^3^Department of Zoology, Cholistan University of Veterinary and Animal Sciences, Bahawalpur, Pakistan; ^4^Faculty of Biosciences, Cholistan University of Veterinary and Animal Sciences, Bahawalpur, Pakistan; ^5^Institute of Biochemistry and Biotechnology, University of Veterinary and Animal Sciences, Lahore, Pakistan; ^6^Faculty of Veterinary Science, University of Agriculture, Faisalabad, Pakistan; ^7^Department of Clinical Laboratory Sciences, College of Applied Medical Sciences, King Khalid University, Abha, Saudi Arabia; ^8^Department of Biology, College of Science, University of Hail, Hail, Saudi Arabia

**Keywords:** nanoparticles, oxidative stress, neurotoxicity, hepatotoxicity, skin sensitization, plant antioxidants, coupling agents, stabilizing agents

## Abstract

Nanoparticle applications are becoming increasingly popular in fields such as photonics, catalysis, magnetics, biotechnology, manufacturing of cosmetics, pharmaceuticals, and medicines. There is still a huge pile of undermining information about the potential toxicity of these products to humans, which can be encountered by neuroprotective antioxidants and anti-inflammatory compounds. Nanoparticles can be administered using a variety of methods, including oronasal, topical applications, and enteral and parenteral routes of administration. There are different properties of these nanomaterials that characterize different pathways. Crossing of the blood-brain barrier, a direct sensory nerve-to-brain pathway whose barriers are bypassed, these checks otherwise prevent the nanoparticles from entering the brain. This inflicts damage to sensory neurons and receptors by nanoparticles that lead to neurotoxicity of the central nervous system. A number of routes make nanoparticles able to penetrate through the skin. Exposure by various routes to these nanoparticles can result in oxidative stress, and immune suppression triggers inflammatory cascades and genome-level mutations after they are introduced into the body. To out-power, these complications, plant-based antioxidants, essential oils, and dietary supplements can be put into use. Direct nanoparticle transport pathways from sensory nerves to the brain via blood have been studied grossly. Recent findings regarding the direct pathways through which nanoparticles cross the blood-brain barriers, how nanoparticles elicit different responses on sensory receptors and nerves, how they cause central neurotoxicity and neurodegeneration through sensory nerve routes, and the possible mechanisms that outcast these effects are discussed.

## 1. Introduction

Nanotechnology is a new scientific field that studies minerals with particles between 1 to 10 microns in size (Tammam et al., 2020). Nanoparticles possess exceptional therapeutic effects when they hold an interaction with biological molecules due to their nanometer particle sizes. It is believed that nanoparticles are more beneficial than mass materials because of their small surface-to-volume ratios, high reactivity, solvency, and bioactivity, as well as controlled molecule size, site-specific focusing and bioavailability (Youssef et al., 2020). Nanoparticles are now being widely used in biomedical applications like anticancer (El-Dawy et al., 2022) which is now being reported in feed supplementation for better quality meat (Khan et al., 2022; Samy et al., 2022). There is a wider range of nanoparticles based on their preparation e.g., green synthesized nanoparticles like turmeric nanoparticles (Sarwar et al., 2021), green synthesized silver nanoparticles (Jalil et al., 2021), and chemical synthesized nanoparticles (Aymen et al., 2022) against different pathogens. The application of nanoparticles is now being evaluated in fish (Aziz et al., 2021, 2022). Recently, nanoparticles are also used against ticks (Kandeel et al., 2022; Zaheer et al., 2022). The chemical properties of these compounds make them useful in cosmetics (Wiechers and Musee, 2010), biotechnology (Singh and Nalwa, 2011), as nanofillers and nanosensors used for the remediation of environmental pollution (Li et al., 2008). Their mass-scale applications have made biological life and humans more exposed to the increased risk of metallic NPs. They may get absorbed into the biological bodies and are redistributed to secondary targets post-exposure. There has been extensive *in vivo* research that shows metal base nanoparticles may have toxic effects when administered intravenously (De Jong et al., 2008), orally (van der Zande et al., 2012), or intraperitoneally (Daniel et al., 2010). The vital and visceral organs; the brain, liver, lung, kidneys, and spleen are among the organs which are likely to come across, absorb and interact with them. In spite of being the chief organ in the body, the brain is highly susceptible to the noxious effects of metallic nanoparticles (Feng et al., 2015). The list of effects that are caused due to metallic nanoparticle neurotoxicity includes; oxidative stress (OS), apoptosis, autophagy, inflammation, and disturbed sensory-motor signaling pathways. Cerebral tissue is principally composed of lipids, and brain oxygen consumption accounts for nearly half of the total body’s consumption of oxygen. Due to its sensitivity to hypoxic injury and oxidative damage, the brain is more susceptible to hypoxic and ischemic tissue damage.

The nervous system is broadly divided into central and peripheral innervations. Neuron cells (neuron body) and their processes (axons and dendrites) that make up the peripheral nervous system (PNS) transmit information to and from muscles, glands, sense organs, and the spinal cord or brain. Peripheral and cranial nerves contain bundles of nerve fibers formed by PNS axons that are sheathed by Schwann cells. The somatic (voluntary) and visceral (automatic) components of the nervous system are made up of afferent (sensory) and afferent (motor) fibers, respectively. Unlike visceral afferent fibers, which carry impulses from the intestines, heart and blood vessels, glands, and various organs, somatic afferent fibers carry information from specific sense organs and sensory receptors in the skin and muscles to the brain. Striated muscles are supplied by somatic afferents, while smooth muscles in the heart, intestine, blood vessels, and glands are supplied by visceral afferents. Peripheral neuropathies due to sensory loss (e.g., loss of sensitivity to vibration, touch, or body orientation) and motor (muscle) weakness result from degeneration of sensory and motor fibers in toxic PNS states. Abnormal sweating, cardiovascular changes, or disorders of the gastrointestinal tract, urinary tract, genitals, or other organs or systems may result from the breakdown or dysfunction of autonomic fibers (Sánchez-Rodríguez et al., 2011).

Different factors determine how nanoparticles affect biosystems. According to their shape, size, and interaction with tissues, nanomaterials can be toxic. In the body, NPs may cause phagocytic cells to “overload,” resulting in a defensive fever and depression in immunity. The inability of organs to effectively degrade NPs may cause them to accumulate. The large surface area of NPs makes them capable of causing enzymes and proteins to dysfunctional, thus dysregulating the cellular-level biological processes. The regeneration of neurons may not be possible as it is for other damaged tissues of many organs involved in active metabolism. Since most of the drugs are unable to pass the BBB in the brain, rendering them from the ability to affect the neurons. Thus, it is particularly important to evaluate nano-neurotoxic effects comprehensively and systemically for preventing or reducing CNS damage. Long-term exposure to Nano titanium (n- TiO 2) NPs inhibits ERK signaling and produces ROS, which can interfere with both mitotic progression and chromosomal segregation. It has recently been proposed that AgNPs directly interact with membrane receptors, causing ROS to be produced and activating signaling pathways involving protein kinases (Marano et al., 2011). The genes that activate these pathways, nuclear factors, or specific genetic programs are dependent on ROS production intracellular and extracellular, despite their varying chemical patterns and differential activities (Nel et al., 2009). NPs have been shown to affect the CNS and the possible mechanisms for these effects are discussed in this review.

## 2. Routes of exposure

The most usual way of exposure to nanoparticles is via oral intake or a central route (Sohal et al., 2018). However, the absorption of nanomaterials through the dermal route has usually been determined to be non-significant (Filon et al., 2015). Nanomaterials also come in contact by parenteral routes and directly with skin, such as topical applications; cosmetics or sunscreens. By the intra-nasal route, particles inhaled are absorbed widely, while the particle density and size contribute to the pattern of particle deposition. The olfactory bulb or other brain sensory receptors are believed to receive particles through neurons in the trigeminal nerve or olfactory bulb (axonal transport) also by paracellular pathways. A mucous membrane in the head region, such as the nasal cavity is impinged by these nerves (Bourganis et al., 2018). The transport across the blood-brain barrier, makes them reach the central nervous system without getting absorbed into the bloodstream. Pulmonary macrophages can also inculcate the inspired particles in the trachea and bronchi, either transporting them to the lymphatic system or removing them from the mucociliary escalator to be phagocytosed by the mucosal cells. In addition to penetrating the alveolar space of the lung, nanometer particles can also dissolve ionic particle components and translocate into the bloodstream. Lastly, particles that were consumed by ingestion of food and beverages may get excreted through lung clearance mechanisms. It is possible that a small percentage of these nanoparticles may enter the digestive system and become toxic via the esophageal route. Nano- titania (n-TiO 2) used in the food industry (as a whitening agent) is also absorbed in the body. Inside brain, particles may enter via passing through the blood-brain barrier due to systemic absorption.

NPs are absorbed through three primary pathways in humans: the respiratory tract (especially the nasal passages), the digestive tract, and the skin. The blood-brain barrier (BBB), blood-cerebrospinal fluid barrier (BCSFB), and blood-nerve barrier (BNB) are the three barriers through which NPs can enter the nervous system. A direct route to the brain is provided by exposed cranial nerves (see VI.D. Direct uptake into the brain through exposed cranial nerves). There has been evidence that metal-based nanoparticles are absorbed from the mouth cavity, the portions of the large intestine; the rectum and cecum, but not from the colon. There is no evidence of sublingual NPs absorption. Absorption by the stomach and intestine depends on the size of the NPs (Hillyer and Albrecht, 2001). Nano- titania (n- TiO 2) has been investigated most extensively for cutaneous absorption because it is extensively used in sunscreen products. Despite the limited detailed evidence, metal-based NPs can be absorbed into the body after dermal application, especially when skin injury, organic solvents, or irritating detergents are not present.

### 2.1. Olfactory nerve pathway

The thousands of olfactory sensory nerves that line the olfactory epithelium form an olfactory nerve pathway through the cribriform plate and olfactory bulb, allowing these nano particles to access the brain (Moseman et al., 2020). In order to ensure the morphological and physiological balance of the human brain, which coordinates almost all body systems, a strict morphological and physiological balance is essential. Through its influence on a key interaction between the nervous system and the environment, the nose-brain interface regulates the immune activity and fluid clearance. It acts as a major physical barrier against pathogenic organisms’ making a difficult entrance into the central nervous system (CNS) by mediating the physiological protection of the brain. Cerebrospinal Fluid (CSF) circulation is another function of the nose-brain link, in addition to regulating environmental microorganisms. A neurodegenerative process can occur in the brain when nanoparticles are inhaled from air pollution and occupational exposure (Calderon-Garciduenas et al., 2002). The olfactory bulb is believed to be a possible passage for metal nanoparticles to migrate into the brain after depositing in the nasal cavity’s olfactory area. From inhaled particles, nanoparticles reach the brain, but how they do so is undercover.

It has been found that the pathway from nose to brain works in most primates (Dorman et al., 2006), but not in humans. It has been suggested that manganese particle transfer from the nose to brain may contribute to Parkinsonian-like symptoms in welding fume workers (Antonini et al., 2006). Quantifying the number of nanoparticles inhaled by humans and rats is crucial in the investigation of this potential exposure route. For nanoparticles deposited in the human olfactory area, inhalation rates ranged from 15 to 30 L/min and particle sizes varied from 1 to 100 nm. In small particles between 1 and 2 nm that are inhaled, our models estimated that around 1% accumulates in the olfactory area. The nasal epithelium covers a larger portion of the nasal surface in rats, and their nasal tubes are smaller than in humans, which may explain the less effective olfactory deposition in rats. Despite a larger minute volume in humans, humans have a higher olfactory dose per unit surface area than rats between 1 and 10 nm, which is consistent with the increased minute volume.

### 2.2. Trigeminal nerve pathway

Olfactory nerves are exposed to the environment only at their ends in the nose and the trigeminal nerve can be found in the oral cavity and roof of the mouth. Colloid silver-coated gold at 50 nm was shown to absorb into the nasal passages, olfactory bulb, and across synapses to connect neurons in the brain. A brainstem’s pons is its biggest and most important region, where these three sensory nuclei converge. The trigeminal ganglion develops on either side of this sensory root as it exits the brainstem. Using nasal medication as a method of crossing the blood-brain barrier offers an intriguing way to transport across the barrier, as olfactory and trigeminal neurons transmit drug molecules directly to the brain. It remains unclear, that whether intact nanoparticles can be transported from nose to brain and along which sort of pathways. Nanoparticle behavior was monitored using accumulation-caused quenching probes, which allow fluorescence switching between loaded and released states. *Ex vivo* histological examinations of rats following nasal injection showed evidence of intact nanoparticles and Cur being transferred. Although preserved PCL nanoparticles cannot penetrate the nasal canal, free Cur molecules released by the nanomaterials can. The mucosa and the trigeminal nerve can be penetrated by PCL NPs and PEGylated PCL NPs containing Cur. Trigeminal neurons are less likely to absorb NP after PEGylation, despite the fact that PEGylation promotes NP retention and mucus penetration. NPs move slowly along the trigeminal nerves. Within an hour of injection, the brain was no longer containing Cur signals or carriers. Two hours after injection, Cur-loaded PEGylated PCL Nanoparticles were present in the brainstem, indicating both intact nanoparticles and Cur were delivered. Once the NPs reach the brain they can travel to other parts of brain, such as mid and forebrain. Other than the olfactory nerve, intact polymeric nanoparticles are primarily transported from the nose to brain via the trigeminal nerve channel (Li et al., 2019).

### 2.3. Blood-brain pathway

Nanoparticles chiefly may not be able to reach the brain because of Blood-Brain Barrier (BBB) and clearance processes through metabolism, distribution, and excretion. An important component of NPs removal from circulation is by mononuclear phagocytes. In order to increase Nanoparticle transport to the nervous system, NPs dosage may need to be increased. In addition, this raises concerns about the long-term effects of permanent nanoparticles because it increases the possibility of undesired outcomes and increases the load on the Nanoparticle system. The NPs can also be made to escape the mononuclear phagocyte system by using PEG and other surface modifications. It is possible to modulate the surface to facilitate selective uptake into the target site, such as by utilizing a synthesis process that is only recognized by tumor tissues when treating that situation, use magnetic nanoparticles directly aimed at the target brain site using external field targeting, and open up the BBB for a short period of time. The blood–brain barrier (BBB) has a unique barrier function that is created by endothelial cells in brain capillaries, pericytes, and astrocytes, which are part of the neurovascular unit, and protects the brain from potentially harmful endogenous and exogenous substances. Efflux transporters and tight junctions in the BBB prevent most therapeutic agents from entering (Bernacki et al., 2008).

A promising approach to overcome limited flux into the central nervous system (CNS) is the use of nanoparticles. Due to their nano size and target specificity, nanoparticles are able to cross the blood-brain barrier and cross the tight junctions of the brain (Agarwal et al., 2009).

**FIGURE 1:**
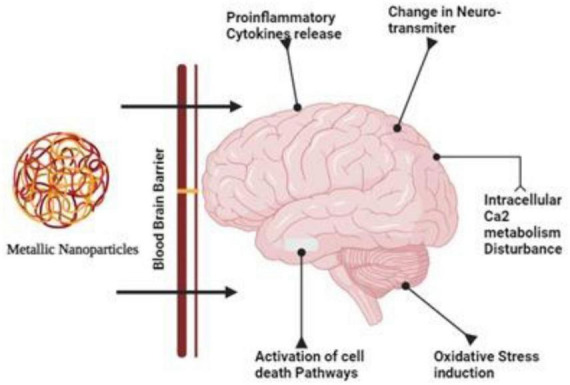
Due their smaller size and target specificity nanoparticles can cross the tight junctions of the brain and cause disturbance in the calcium metabolism and activates necrosis, apoptosis and bring changes in the neurotransmitters.

A greater understanding of nanoparticles’ effects on the BBB and CNS is needed, however. Ag-NPs have been shown to cross the BBB *in vitro* and *in vitro*, causing BBB dysfunction and neurotoxicity in recent years (Tang et al., 2009; Figure 1).

### 2.4. Exposure through ingestion and inhalation

Food components, supplements, and additives are being synthesized using nanoparticles in the food industry in order to improve nutrient absorption, better packaging, and enhance nutrient absorption capacity (Sufian et al., 2017). There are also non-edible items that may contain nanoparticles, such as food and drink containers, and surfaces with silver nanoparticles (Warheit and Sayes, 2015). There are some safety risks associated with these technologies, despite their positive impacts (Lundquist and Artursson, 2016). It is recommended to administer drug-encapsulated nanoparticles, which boost bioavailability via multiple mechanisms, through the oral route because of their simplicity (Date et al., 2016). After ingestion, nanoparticles may not reach their target cells because of various barriers, such as the stomach and intestinal milieu, the mucus barrier, tight junctions limiting paracellular transit, and the epithelial cells lining the digestive tract (Lundquist and Artursson, 2016). Therefore nanoparticles are absorbed through paracellular absorption (passing between cells) as well as transcellular uptake (attached to specific receptors or passed passively through enterocytes (Momin et al., 2013). Accordingly, nanoparticles can pass through the intestinal system and enter the circulatory system depending on their size, dispersibility, and charge (Warheit and Sayes, 2015). Nanoparticles smaller than a nanometer be transported by the blood-brain barrier and become trapped in the brain (Caito and Aschner, 2015).

A significant danger associated with inadvertent consumption of nanomaterials is because they are widely used in fabric, paint, beauty products, water cleaning agents, and packaged food (Geiser et al., 2017). Nanoparticles with diameters of 1–5 nm get accumulated in the upper airway area, and larger particles with diameters of 0.1–1 nm enter the alveoli, depending on the size. Larger particles with diameters of 5–30 nm stay in the nasopharyngeal area, and smaller ones with diameters of 0.1–1 nm go into the alveoli. By contrast, nanoparticles with a size less than 0.5 nm can pass through the thin epithelium to reach the blood capillaries (De Matteis, 2017). The nasal passages have been discovered to contain nanomaterials, which can either enter the capillaries beneath the respiration epithelium or be digested by the olfactory system and enter the brain (Fröhlich and Salar-Behzadi, 2014). Although non-therapeutic forms of nanoparticles are the most common subject of toxicological studies, most of these lead to morbidity or death. In contrast to therapeutic nanoparticles, these nanoparticles are significantly smaller, inorganic, and insoluble in water, and require different dosages and dosing frequencies. Therefore, nanomaterials used in biological systems could be subjected to toxicity studies (Zhang J. et al., 2011).

### 2.5. Exposure through skin contact and systemic intake

Skin contains both lipophilic and hydrophilic substances, so it may acquire both through different pathways. As a result of their physicochemical characteristics, nanoparticles are capable of infiltrating the skin through a number of pathways, facilitating passage into the systemic circulation (Teixeira et al., 2018). The epithelium, corneocytes, and hair cells absorb small nanoparticles intracellularly, intercellularly, and dermally (Palmer and DeLouise, 2016). Biomedical nanoparticles’ size and ionizing potential determine their bioavailability, and skin integrity influences the substance’s absorption. In spite of this, toxicology studies suggest that only certain types of nanoparticles penetrate and permeate the skin (Mauro, 2018).

It is mostly associated with nanotechnology that nanoparticles are exposed via intravenous infusions (Warheit and Sayes, 2015). In medicine, they are used to diagnose and treat a wide range of illnesses. The use of nanoparticles that can pass through the bloodstream is necessary for the detection and treatment of brain cancer and central nervous system disorders.

## 3. Mechanisms of damage to the brain

It has been demonstrated that NPs transported along sensory nerves to the brain are capable of targeting the olfactory bulb, cortex, striatum, hippocampus, cerebellum, and brainstem (Hemmink et al., 2016). As sensory nerve-to-brain pathways pass through the brain, nanoparticles are deposited in different areas. As NPs pass through the olfactory nerve and reach the brain, they are deposited in the olfactory bulb in significant quantities (Kwon et al., 2013). Most NPs transported via the taste nerve-to-brain pathway are directed toward the cortex (Liang et al., 2018). An interesting finding was that the nanoparticles were initially located in the same area as the target area. The olfactory nerve is a pathway nanoparticles take to enter the brain in an investigation by Kim et al. (2016). Following intranasal administration of gelatin nanoparticles (GNPs), brain regions including the olfactory bulb, cortex, and striatum were revealed to have been affected within one hour, which confirms previous research on GCPQ nanoparticles. Researchers found that NPs are primarily transported through the olfactory bulb before reaching the cortex and thalamus, according to Godfrey et al. (2018).

### 3.1. Organelle damage

**FIGURE 2:**
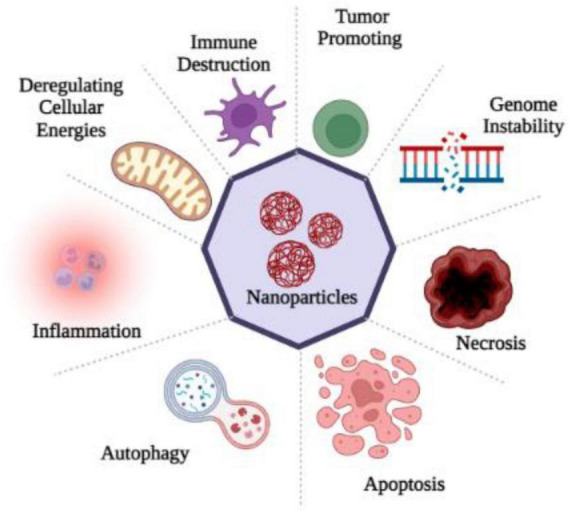
Nanoparticles entry into the human brain through various routes of exposure produced ROS which causes oxidative stress and as a consequence of it inflammation, apoptosis, necrosis, gene disability, immune destruction and the dis-regulation of cellular energies occur, that disturbs the cell homeostasis.

Through sensory nerve pathways, nanoparticles can induce a variety of ultrastructural changes in the brain, including mitochondrial dysfunction and nuclear damage. The intranasal administration of TiO2 nanoparticles to brain tissue leads to a significant alteration in the nuclear membrane, chromosome marginalization, and mitochondrial swelling (Ze et al., 2014). The installation of ZnO and TiO2 NPs in the brain also resulted in mitochondrial swelling and fragmentation (Aijie et al., 2017), as well as a karyopyknotic and a karyorrhexic appearance. A more detailed description of mitochondrial injury caused by copper nanoparticles was also provided by Liu et al. (2014). As a result of mitochondrial aggregates in the olfactory bulb, less ER organelles were present and ER ribosomes became dissociated (Liu et al., 2014). The lysosomes might be another target, in addition to mitochondrial impairments and nuclear defects. As a result of an intranasal instillation, Fe_2_O_3_ nanoparticles are transported to hippocampal lysosomes. Oxidative stress is closely associated with mitochondria (Sharma et al., 2018), whereas autophagy is associated with lysosomes (He et al., 2013). Therefore, neurotoxicity might be linked to oxidative stress and autophagy mechanisms (Figure 2).

### 3.2. Oxidative stress

**FIGURE 3:**
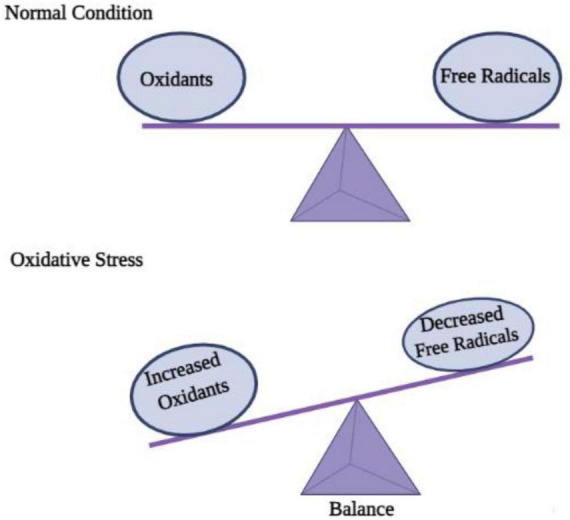
Disequilibrium between oxidation and free radicals disturbs cell homeostasis and causes oxidative stress.

ROS (reactive oxygen species) contain oxygen atoms and can be highly reactive. Examples would be superoxide radicals and H_2_O_2_ (Rang and Boonstra, 2014). As a result of mitochondrial and cytoplasmic oxidation, ROS are present in every cell under physiological conditions. Normal physiological processes require low to moderate ROS concentrations. It would be extremely damaging, however, to produce an excessive amount of ROS as a result of oxidative stress. Since the CNS consumes so much oxygen, has weak antioxidative abilities, and has terminal differentiation, it is highly susceptible to oxidative stress (Li et al., 2013). The oxidative stress associated with acute-CNS-injury-related neurodegenerative diseases is known to cause DNA damage that impairs cerebral cell viability (Smith et al., 2013). The role of ROS in neurodegenerative diseases such as Alzheimer’s has been repeatedly demonstrated in studies. Apoptosis of neurons is also an important mechanism in brain dysfunction, which is mediated by ROS (Sorce and Krause, 2009). As well as regulating neuronal ion channels, transcription factors, and kinases, ROS can alter the genetic code of neurons (von Bohlen und Halbach, 2007). In addition to contributing to long-term memory dysregulation, ROS generated by the NADPH oxidase 2 (Nox2) system also contributes to it (Massaad and Klann, 2011). ROS damages membranes, denaturing lipids and altering DNA structures, as well as altering the structure and function of internal proteins. A mutation or alteration in gene expression can result from DNA oxidation, which is extremely concerning. In mitochondrial DNA, mutations caused by ROS are more likely to occur because there are no DNA repair enzymes. Protein oxidation may result in the accumulation of insoluble proteins in some diseases, including neurodegenerative diseases (Brieger et al., 2012). Due to their large surface areas, nanoparticles may cause cytotoxicity due to their ability to generate ROS, a prime factor in disease progression and cellular stress (Nel et al., 2006). Contrary to this, it is unclear how the central nervous system is affected by NP-induced ROS. Numerous nanomaterials have been shown to induce excessive levels of ROS in cells, including quantum dots and metal-oxide nanoparticles (Hanley et al., 2009; Figure 3).

A major cause of poisoning is excessive ROS production caused by nanoparticles transmitted through sensory nerves to the brain (Ema et al., 2016; Tapeinos et al., 2017). It has been shown that excessive ROS production by Nano- titania (n- TiO 2) nanoparticles causes brain diseases, as shown by the impairment of memory recognition after exposure (Czajka et al., 2015). Due to nanoparticles, the excessive production of ROS is also accompanied by the production of other oxidants and reduced antioxidant activity. There was a significant increase in malondialdehyde levels, SOD levels, hydrogen peroxide levels, and GSH activity among the nanoparticles intranasally instilled (Liu et al., 2014), Fe_3_O_4_ nanoparticles (Wu et al., 2013), and ZnO nanoparticles (Liu et al., 2009). Nanoparticles containing ZnO and TiO2 were found to reduce the brain’s levels of SOD, GSH, glutathione peroxidase (GSH-Px), and GSH/glutathione disulfide (GSSG), whereas MDA levels were increased (Aijie et al., 2017). Aside from this, the authors of the same study noted that Cyp51 and Gsr genes were upregulated, whereas Nqo1, Fmo2, and Dhcr7 genes were downregulated (Aijie et al., 2017). There may be a mechanism that induces brain toxicity after NPs pass through sensory nerve pathways.

### 3.3. Inflammation

Inflammation is the body’s response to phagocytosis, which is followed by several immune regulatory molecules. Various studies have revealed that carbon nanotubes and fullerene derivatives induce inflammation in different types of cells, including alveolar epithelial cells, epidermal keratinocytes, and monocyte macrophages cultured *in vitro* (Baktur et al., 2011). In response to oxidative stress, proinflammatory mediators are released through nuclear factor-B, mitogen-activated protein kinase (MAPK) and phosphoinositide 3-kinase (PI3-K) pathways (Poljak-Blaži et al., 2010). This indicates that oxidative stress and inflammation are mutually interconnected (Allen and Tresini, 2000). In addition to zinc, cadmium, silica, and iron nanoparticles, many metal oxide nanoparticles have been shown to be toxic by triggering inflammatory cytokines (Pujalté et al., 2011). Several cellular processes are regulated by the MAPK pathway, including cell division, proliferation, mitosis, survival, and apoptosis (Torres and Forman, 2003).

### 3.4. Apoptosis

**FIGURE 4:**
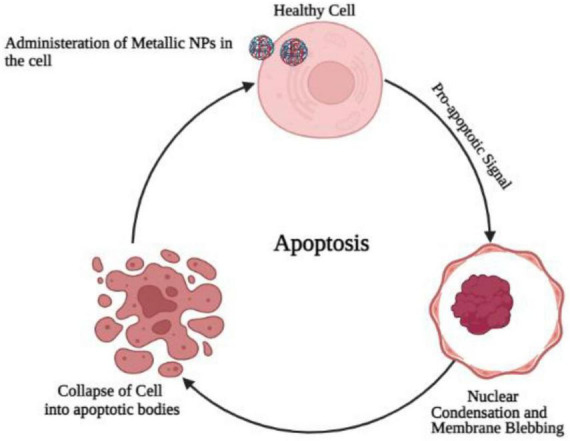
Administration of metallic oxide nanoparticles in the healthy brain cells causes programmed cell death due to the increase production of oxidative stress and convert normal cell into cancerous cell.

One of the most commonly studied PCD types is apoptosis. The phenomenon can be defined simply as programmed self-destruction (Maiuri et al., 2007). An important role of apoptosis is to renovate cells as well as to eliminate those that have been injured. A malfunctioning apoptotic process can cause cell death and tissue damage, resulting in organ dysfunction (Elmore, 2007). An important aspect of human health and disease is the ability of cells to undergo apoptosis (Wirawan et al., 2010). Blebbing, fragmentation of DNA, and activation of caspases are some of the hallmarks of apoptosis (Kanter et al., 2016). Although apoptosis played a role in their neurotoxicity, metallic nanoparticles do not seem to regulate apoptosis by regulating apoptosis. Cells are believed to apoptose as a result of oxidative stress (OS) (Hildeman, 2004). In order to verify that NP-induced OS leads to neurotoxicity, rescue studies were conducted. As a result of exposure to TiO2 NP, PC12 cells had diminished viability, increased ROS production, and showed an increase in apoptotic cells. It is possible to reverse these changes, however, if PC12 cells are treated with N-acetylcysteine (NAC). This suggests that ROS generated by TiO2 NPs are responsible for PC12 apoptosis (Liu et al., 2010; Figure 4).

### 3.5. Autophagy

**FIGURE 5:**
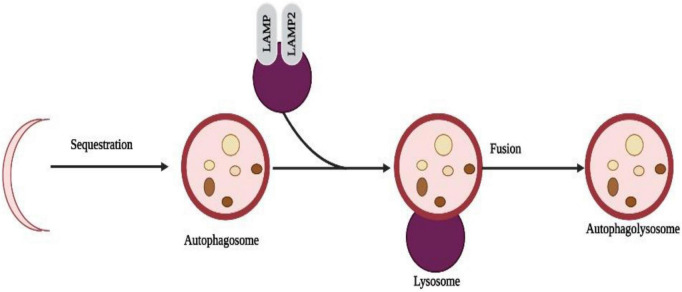
Selective sequestration of invasive microbes occur through the automacrophagy nanoparticles entry into cell speeds up the self-eating process and destroys the normal cells with pathogenic cells.

Recently, autophagy has gained much attention and has become a hot topic. Programmable self-eating can be described simply as this concept (Maiuri et al., 2007). An autophagic process differs from an apoptotic process by relying on caspase-independent pathways. Starvation adaptation is a process that occurs in cells in response to starvation. When cells are degraded, their cargo is transported to lysosomes, a major component of autophagy. Non-neuronal cells, such as human keratinocytes, can be induced to undergo autophagy by metallic nanoparticles (HaCaT) (Mizushima and Komatsu, 2011; Lopes et al., 2016). Nanotoxicity has been linked to autophagy (Cohignac et al., 2014) as one mechanism of actions. A study in human cerebral endothelial cells (HCECs) revealed the presence of autophagic vacuoles after attachment of aminoPVA [poly(vinyl alcohol/vinylamine)]-coated USPIO NPs. Also, NPs increased cathepsin D protein levels in HCECs, suggesting autophagy is induced by them (Kenzaoui et al., 2012; Figure 5).

## 4. Nanoparticles’ effects peripheral nervous system

It is possible that NPs might alter sensory neuroreceptor morphology since they induce toxicity within sensory neuroreceptors. Due to this, NP effects on sensory neuroreceptor morphology have been rarely studied *in vivo*, with imprecise descriptions provided. Hyperemia of the fungiform papilla occurs when ZnO nanoparticles are injected under the tongue (Liang et al., 2018). In addition, intravitreal nanogold injections induce mild vacuolization in ganglion cells which disrupts outer photoreceptor segments. It is still necessary to conduct more research on the sensory organs and their sensory neuroreceptors. Acute inflammation is observed in the eyes of mice after intravitreal injection of AuNRs (Gabriele Sandrian et al., 2012). Inhalation of CuO nanoparticles causes the olfactory epithelium to degenerate (Gosens et al., 2016). There is an interesting difference between the effects of NPs and ions in the solution. Intranasal administration of ZnO nanoparticles to rats resulted in olfactory epithelium edema, cluttered epithelial columnar cells, sparse cell layers, and shrinkage. Nanoparticles of ZnO suspended in PBS, however, do not cause significant harm (Gao et al., 2013). Further, we found that ZnO NPs increased intracellular Zn ions, resulting in cytotoxicity and olfactory dysfunction, rather than Zn ions eliciting olfactory epithelial toxicity (Qin et al., 2017). *In vitro* studies may provide some insight into how NPs affect the morphology of sensory neuroreceptors, particularly when it comes to the non-metallic NPs. It is known that the dose of NPs has a significant impact on sensory neuroreceptor morphology. As a result of the LNCs, cochlear cell morphology is disrupted and there is an increase in apoptosis or necrosis (Zhang Y. et al., 2011). It is also important to note that the size of NPs plays a role in affecting neuroreceptor morphology in addition to the dose. NPs with a diameter of 20 nm destroyed the epithelium layer of porcine olfactory epithelium *in vitro*, whereas NPs with a diameter of 100 nm disturbed its integrity.

### 4.1. Effects on the morphology of the neuron receptors

**FIGURE 6:**
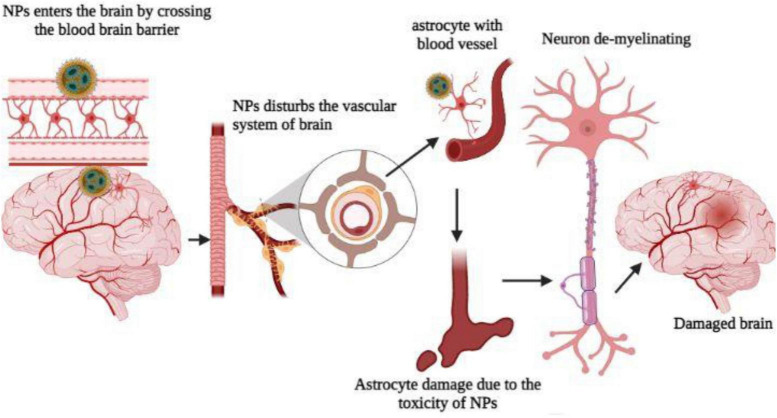
Nanoparticles affect the brain and damage the neuron.

A neuron can be classified into three major morphological groups depending on how many dendrites it has (i.e., its primary dendrites); this classification system applies to all animals and plants. In multipolar neurons, there are more than one primary dendrite, similar to what is found in mammalian pyramidal neurons. On the other hand, bipolar neurons have a single primary dendrite from which large dendritic arbors may emerge (e.g., cerebellar Purkinje cells) or may not arise (e.g., photoreceptors). An important point to note is that unipolar neurons, such as DRG neurons in vertebrates and most CNS neurons in invertebrates, usually extend only one neurite. Since the dendritic arbor has a multipolar morphology, several distinct fields surround its soma, which affects not only how passive current is distributed in neurons and how electrical signals are processed, but also what type of synaptic or sensory inputs neurons receive (Spruston and Johnston, 2008). Neurons have three primary morphologies, which serve as both explanations for differences in organization principles between species of animals and within the same species. Vertebrates have multipolar neurons in contrast to invertebrates that have unipolar ones (Grueber et al., 2005). Although all three morphological types have been found throughout evolution, most invertebrates have unipolar neurons. Monopolar neurons originate from the soma of insects and project their somatic processes to synapse-enriched neuropils. They then divide into dendrites, which arborize locally, and axons, which extend into other neuropil areas. Due to the unipolar organization of neuronal processes, synaptic connections can be formed away from the cell body of the neuron, suggesting that neuronal migration is not uncommon in the insect CNS (Harris and Fallot, 2001), but is common in vertebrates. It has taken almost a century for the molecular and cellular mechanisms that lead to postmitotic neurons developing multipolar, bipolar, or unipolar morphologies to be understood, despite the importance of these basic neuronal organizations. The functioning of astrocyte cells was impacted by titanium dioxide nanoparticles, the second-most prevalent nanomaterial in the world. Astrocytes serve a variety of roles, including providing energy to the neurons that process signals and regulating the exchange of neurotransmitters that carry signals in the brain. Glutamate is a neurotransmitter that normally enters and is processed by astrocytes and has a variety of functions in cognition, memory, and learning, as well as the development, migration, and maintenance of other cells. Glutamate, however, turns into a lethal toxin when it builds up outside of cells, increasing the risk of neurodegenerative illnesses like Alzheimer’s and Parkinson’s (Kofroñová et al., 2020; Figure 6).

### 4.2. Effects on the receptor function

**FIGURE 7:**
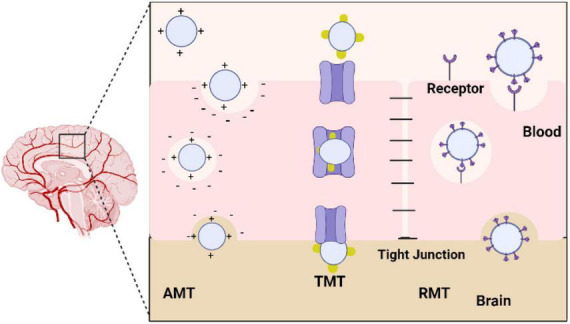
Diagram showing the various methods used by nanoparticles to cross the blood-brain barrier (BBB). AMT, adsorptive-mediated transcytosis; TMT, transporter-mediated transcytosis; RMT, receptor-mediated transcytosis.

Humans can be exposed to metallic oxide nanoparticles from different sources, including the environment and the workplace. NPs can be produced by natural processes, such as volcanic activity, and by industrial processes, such as cutting, grinding, melting, casting, and welding. The use of metallic nanoparticles in food products is in addition to their intentional use in pharmaceutical vectors, sunscreens, toothpaste, cosmetics, plastics, textiles, and paints. There are several methods by which NPs can enter the body, including injections, inhalations, and ingesting, despite the fact that they originate from different sources. Once they reach the bloodstream, they penetrate and accumulate in several tissues and organs, including the central nervous system (CNS) (Chen et al., 2008). When developing solutions to meet this need, active targeting is especially crucial because it enables the targeted delivery of drugs to the brain, their site of action, by guiding nanoparticles to the desired location. In fact, the surface area to volume ratio of these nano systems is very high, allowing the nanoparticles to be highly chemically reactive and allowing surface modification with molecules that may be recognized by receptors/transporters overexpressed in the BBB and cell-specific receptors in the brain tissue. Adsorptive-mediated transcytosis, transporter-mediated transcytosis, and receptor-mediated transcytosis are essentially three different methods for achieving this goal. Nanoparticles must be able to access the target once inside the brain, such as brain tumor cells, neurons, or even the fibrils linked to many neurological disorders (Kofroñová et al., 2020; Figure 7).

## 5. Cope up strategies

### 5.1. Plant-based antioxidants

In response to stress conditions, plant antioxidants are generally activated and biosynthesized to prevent oxidative damage caused by ROS. There was an interesting observation that plant stress tolerance differs between sensitive and tolerant cultivars of the same species according to how well they were able to counteract oxidative stress by increasing antioxidant enzyme activity and biosynthesis. The effects of salt stress on tomato (*Solanum lycopersicum*) were elevated superoxide and H_2_O_2_ levels, as well as increased lipid peroxidation. These responses led to an increase in the activity of SOD, CAT, and enzymes involved in the AsA-GSH cycle (APX, MDHAR, DHAR, and GR). In addition, salinity caused significant increases in AsA, GSH, and carotenoid concentrations. As a consequence of the latter observation, it appears that ROS are damaging photosynthetic machinery. In contrast to cultivated tomatoes (*S. lycopersicum*), wild tomato relative **Solanum chilense** suffered less ROS-induced damage during salt stress, which is likely due to a stronger activation of the antioxidant machinery in the former species when salt was applied (Sánchez-Rodríguez et al., 2011). The antioxidant activity of plants has been demonstrated, using FRAP, DPPH, and ABTS, as well as the ability to scavenge and suppress the formation of (ROS) reactive oxygen species (ROS) (Elghobashy et al., 2020).

Insensitive Solanum species, drought stress reduced the activity of enzymes involved in the shikimate pathway, but not in ones that had learned to tolerate drought stress, which increased the concentrations of phenolic compounds, such as quercetin and kaempferol. It is evident from the study that polyphenols contribute significantly to tomato drought tolerance. Nicotiana tabacum showed significant reductions in chlorophyll and carotenoid concentrations when stressed with drought, while lipid peroxidation and ROS levels showed strong increases. Again, this indicates that the photosynthetic machinery is damaged due to stress. Drought-induced ROS production was countered by significant increases in SOD, POD, APX, CAT, and GR activities, as well as AsA, GSH, and total phenol levels. As a result of inoculating plants with arbuscular mycorrhizal fungi or supplementing them with phosphorus during water deficits, the antioxidant machinery was even more active, emphasizing that microorganisms and soil characteristics influence plant responses to stress (Begum et al., 2020). Tobacco plants are also tolerant to As exposure via antioxidant defense mechanisms, as N tabacum cv ‘Wisconsin’ generally exhibits higher levels of phenolic compounds, AsA, and GSH than Nicotiana sylvestris. There were opposite responses in antioxidant enzyme activity between both genotypes of roots and leaves. There was a decrease in APX, GST, and POD activity in the leaves of the tolerant genotype, whereas there was an increase in the leaves of the sensitive genotype. By contrast, CAT behaved in the opposite manner. There is evidence that antioxidant responses in plants are strongly organ-specific (Kofroñová et al., 2020).

A plant’s antioxidant defense system protects it against a wide range of stress factors, as evidenced by the fact that it is tolerant to a variety of stress conditions when exogenous antioxidants are applied. The antioxidant capacity of plants can also be improved through transgenic methods to improve their stress tolerance. The use of crop engineering techniques to increase crop tolerance to abiotic stresses has been reviewed by Broad et al. (2020). AsA biosynthesis genes can be increased, as can genes responsible for AsA recycling genes, and factors that affect AsA levels can be altered, such as transcription factors controlling genes involved in the AsA biosynthesis pathway (Broad et al., 2020).

### 5.2. Extracts and essential oils

The study found plant essential oils to reduce migraine intensity, attack frequency, and pain by reducing lavender essential oil, peppermint essential oil, chamomile essential oil, anise essential oil, basil essential oil, rose essential oil, and mixed essential oils. Nanoparticles made from plants serve as less toxic and more effective carriers for drugs, thereby improving their bioactivity within tissues and cells (El-Dawy et al., 2022). There is evidence that certain oils can reduce symptoms like photophobia, phonophobia, nausea, vomiting, and other disorders, but there is still much work to be done to determine the specific mechanism. NO and CGRP levels, as well as ET, 5-HT, and c-fos levels, were reduced by Angelicae Dahuricae Radix oil and Chuanxiong Rhizoma essential oil in rats. Inflammation is decreased and abnormal vasomotion may be balanced in the two oils, so they might be able to relieve migraines. A dose-dependent reduction of cortical spreading depression has been demonstrated in the cerebral cortex using garlic essential oil (1–500 mL/L). An inflammatory cascade is triggered by CSD, which causes a slow wave of depolarization to spread rapidly among neuronal and glial cells. In addition, it activates the NF–B pathway in astrocytes. Researchers have found that garlic oil inhibits neurogenic inflammation and central sensitization, which might alleviate migraine symptoms (Yang et al., 2015). Researchers have demonstrated therapeutic benefits of nanoformulations of curcumin in the treatment of cancer, cardiovascular disease, and neurological disorders (El-Dawy et al., 2022).

### 5.3. Dietary supplements

Cellular neurodegeneration develops years before the clinical manifestations of Parkinson’s disease occur. In order to combat PD, finding strategies that can be applied over an extended period of time would seem logical. Nutrients and functional foods have been found to be neuroprotective against neurodegeneration in an increasing number of studies (Park H. A. et al., 2018). Specific vitamins, minerals, and phytochemicals have antioxidizing properties because they directly scavenge ROS, act as cofactors for antioxidant enzymes, and trigger the production of intracellular antioxidants. With the introduction of advanced liquid chromatography and mass spectrometry technologies such as LC/MS/MS and MALDI-TOF, it is possible to analyze these nutrients quantitatively and apply molecular approaches, such as sequencing, polymerase chain reaction, and electrophoresis, to elucidate the relationship between PARK genes and antioxidants in the diet. PD progression may be delayed or prevented by dietary antioxidants (Park H. A. et al., 2018).

A neurodegenerative disorder called Parkinson’s disease (PD) is caused by loss of dopaminergic neurons in the substantia nigra pars compacta (SNpc). The forebrain substructure that regulates the motor system, the striatum, is also affected by dopamine. Parkinson’s disease is characterized by motor symptoms such as tremor, bradykinesia, rigidity, and speech difficulties, as well as non-motor symptoms such as depression and insomnia (Armstrong and Okun, 2020). Sporadic Parkinson’s disease is caused by a number of factors, such as lifestyle, environment, and age. Neurodegenerative disorders are thought to be caused by a variety of factors, including oxidative stress. ROS-induced PD pathology, in particular mitochondrial dysfunction, is commonly observed (Dias et al., 2013). A complex I enzyme known as NADH oxidoreductase (ETC) assists in oxidative phosphorylation by transferring electrons between NADH and ubiquinone. The damage to complex I caused by oxidative damage creates a positive feedback loop that allows ROS such as superoxide and hydrogen peroxide to be generated. There are currently neurotoxic compounds as well as rotenone that induce oxidative stress *in vitro* and *in vivo* when administered, which trigger parkinsonism models *in vitro* and *in vivo*. In addition, desynaptic neurons are damaged when dopamine metabolism is disrupted, resulting in the production of ROS. Tyramine and tyrosine are amino acids that are necessary to synthesize dopamine in the body. The hydroxylation and decarboxylation of tyrosine lead to the production of dopamine. As a result of this conversion, norepinephrine and epinephrine are produced or dopamine is degraded. It is possible for monoamine oxidase (MAO) to produce 3,4-dihydroxyphenylacetaldehyde (DOPAL), while dopamine itself can be oxidized. It is believed that accumulation of DOPAL and oxidized dopamine damages mitochondria (Park J. S. et al., 2018). The mitochondria can be protected against damage by dietary antioxidants (Park H. A. et al., 2018).

## 6. Impact on human health

In addition to chemical agents, neurotoxicology also examines biological and some physical (such as radiation) agents that have negative effects on the growing, developing, and aging nervous systems, including the neuroendocrine, neuromuscular, and special sense organ systems, as well as on behaviors in both humans and other animals. Some neuroactive substances cause rapidly reversible changes, while others cause permanent damage to the nervous system, and some can cause progressive and eventual degeneration of the nervous system. Substances used (such as alcohol, inhalants and drugs), therapeutic drugs, toxic by-products or components of organisms (such as bacteria, fungi, plants, or animals), chemicals intended to affect organisms undesirable to humans (such as overexposure to fungicides, herbicides and pesticides), industrial chemicals, chemical warfare agents, additive and naturally occurring food ingredients, and some other types of chemicals that are exposed. There is generalized initiation of inflammatory cascades, the mechanisms by which chemicals may cause damage to the neurons. Others cause neurological or behavioral disturbances indirectly, for example by altering electrolyte balance, cerebral blood flow, glucose metabolism, or the levels of key intermediate metabolites. Because of the specific sensitivity of nervous tissue to disturbances in body homeostasis, such pathophysiological changes are often clinically manifested as neurological disorders (Marano et al., 2011). The neuro-pathologies are deeply rooted in the effects of oxidative stress, which may lead to ischemic injuries to CNS. While acute nano particle toxicity may induce, vomiting and convulsions may lead to seizures triggered by hypoxia.

## 7. Conclusion

The recent age of environmental and biological remediating technology has resulted in the vast use of nanoparticles, with underline effects of neurotoxicity. This review article highlights different types of exposure to the metallic nanoparticles which may eventually cause brain damage. Comparatively, phytochemical-based nanocarriers are non-hazardous, environment-friendly, less toxic, easy to manufacture, provide particles in controlled sizes and morphologies, and are inexpensive. A variety of anti-inflammatory compounds in essential oils and antioxidants derived from plants can be used to deal with neuropathologies. There have been reports that NPs are restorative in preclinical models of neurological disorders, but further research is required to address safety concerns. Thus, new research is urgently needed to determine the detrimental effects of nanocarriers on central nervous system, especially their neurotoxicity leading to neuro-degenration and ways to effectively encounter these concerns.

## Author contributions

SZ, AI, and AM conceived the idea, wrote the original manuscript, and revised the manuscript. MF drew the figures. KA, TK, C-NZ, and AS edited the initial draft and revised the manuscript. IA and MS provide financial support and revised manuscript.

## References

[B1] Agarwal A. Lariya N. Saraogi G. Dubey N. Agrawal H. Agrawal G. P. (2009). Nanoparticles as novel carrier for brain delivery: A review. *Curr. Pharm.* 15 917–925. 10.2174/138161209787582057 19275654

[B2] Aijie C. Huimin L. Jia L. Lingling O. Limin W. Junrong W. (2017). Central neurotoxicity induced by the instillation of ZnO and TiO2 nanoparticles through the taste nerve pathway. *Nanomedicine (Lond)* 12 2453–2470. 10.2217/nnm-2017-0171 28972461

[B3] Allen R. Tresini M. (2000). Oxidative stress and gene regulation. *Free Radic. Biol. Med.* 28 463–499. 10.1016/S0891-5849(99)00242-7 10699758

[B4] Antonini J. M. Santamaria A. B. Jenkins N. T. Albini E. Lucchini R. (2006). Fate of manganese associated with the inhalation of welding fumes: Potential neurological effects. *Neurotoxicology* 27 304–310. 10.1016/j.neuro.2005.09.001 16219356

[B5] Armstrong M. J. Okun M. S. (2020). Diagnosis and treatment of Parkinson disease: A review. *JAMA* 323 548–560. 10.1001/jama.2019.22360 32044947

[B6] Aymen N Aqib A. I. Akram K. Majeed H. Murtaza M. Muneer A. (2022). Resistance modulation of dairy milk borne *Streptococcus agalactiae* and *Klebsiella pneumoniae* through metallic oxide nanoparticles. *Pak. Vet. J.* 42 424–428. 10.29261/pakvetj/2022.052

[B7] Aziz S. Abdullah S. Anwar H. Latif F. (2022). DNA damage and oxidative stress in economically important fish, Bighead carp (*Hypophthalmichthys nobilis*) exposed to engineered copper oxide nanoparticles. *Pak. Vet. J.* 42 1–8.

[B8] Aziz S. Abdullah S. Anwar H. Latif F. Mustfa W. (2021). Effect of engineered nickel oxide nanoparticles on antioxidant enzymes in fresh water fish, *Labeo rohita*. *Pak. Vet. J.* 41 424–428. 10.29261/pakvetj/2021.044

[B9] Baktur R. Patel H. Kwon S. (2011). Effect of exposure conditions on SWCNT-induced inflammatory response in human alveolar epithelial cells. *Toxicol. In Vitro* 25 1153–1160. 10.1016/j.tiv.2011.04.001 21477645

[B10] Begum N. Ahanger M. A. Zhang L. (2020). AMF inoculation and phosphorus supplementation alleviates drought induced growth and photosynthetic decline in *Nicotiana tabacum* by up-regulating antioxidant metabolism and osmolyte accumulation. *Environ. Exp. Bot.* 176:104088. 10.1016/j.envexpbot.2020.104088

[B11] Bernacki J. Dobrowolska A. Nierwinska K. Malecki A. (2008). Physiology and pharmacological role of the blood-brain barrier. *Pharmacol. Rep.* 60 600–622. 19066407

[B12] Bourganis V. Kammona O. Alexopoulos A. Kiparissides C. (2018). Recent advances in carrier mediated nose-to-brain delivery of pharmaceutics. *Eur. J. Pharm. Biopharm.* 128 337–362. 29733950 10.1016/j.ejpb.2018.05.009

[B13] Brieger K. Schiavone S. Miller F. J. Krause K. H. (2012). Reactive oxygen species: From health to disease. *Swiss. Med. Wkly.* 142:w13659. 10.4414/smw.2012.13659 22903797

[B14] Broad R. C. Bonneau J. P. Hellens R. P. Johnson A. A. (2020). Manipulation of ascorbate biosynthetic, recycling, and regulatory pathways for improved abiotic stress tolerance in plants. *Int. J. Mol. Sci.* 21:1790. 10.3390/ijms21051790 32150968 PMC7084844

[B15] Caito S. Aschner M. (2015). Neurotoxicity of metals. *Handb. Clin. Neurol.* 131 169–189. 10.1016/B978-0-444-62627-1.00011-1 26563789

[B16] Calderon-Garciduenas L. Azzarelli B. Acuna H. Garcia R. Gambling T. M. Osnaya N. (2002). Air pollution and brain damage. *Toxicol. Pathol.* 30 373–389. 10.1080/01926230252929954 12051555

[B17] Chen L. Yokel R. A. Hennig B. Toborek M. (2008). Manufactured aluminum oxide nanoparticles decrease expression of tight junction proteins in brain vasculature. *J. Neuroimmune Pharmacol.* 3 286–295. 10.1007/s11481-008-9131-5 18830698 PMC2771674

[B18] Cohignac V. Landry M. J. Boczkowski J. Lanone S. (2014). Autophagy as a possible underlying mechanism of nanomaterial toxicity. *Nanomaterials* 4 548–582. 10.3390/nano4030548 28344236 PMC5304698

[B19] Czajka M. Sawicki K. Sikorska K. Popek S. Kruszewski M. Kapka-Skrzypczak L. (2015). Toxicity of titanium dioxide nanoparticles in central nervous system. *Toxicol. In Vitro* 29 1042–1052. 25900359 10.1016/j.tiv.2015.04.004

[B20] Daniel S. Tharmaraj V. Sironmani T. A. Pitchumani K. (2010). Toxicity and immunological activity of silver nanoparticles. *Appl. Clay Sci.* 48 547–551. 10.1016/j.clay.2010.03.001

[B21] Date A. A. Hanes J. Ensign L. M. (2016). Nanoparticles for oral delivery: Design, evaluation and state-of-the-art. *J. Control. Release* 240 504–526. 10.1016/j.jconrel.2016.06.016 27292178 PMC5064878

[B22] De Jong W. H. Hagens W. I. Krystek P. Burger M. C. Sips A. Geertsma R. E. (2008). Particle size-dependent organ distribution of gold nanoparticles after intravenous administration. *Biomaterials* 29 1912–1919. 10.1016/j.biomaterials.2007.12.037 18242692

[B23] De Matteis V. (2017). Exposure to inorganic nanoparticles: Routes of entry, immune response, biodistribution and in vitro/in vivo toxicity evaluation. *Toxics* 5:29. 10.3390/toxics5040029 29051461 PMC5750557

[B24] Dias V. Junn E. Mouradian M. M. (2013). The role of oxidative stress in Parkinson’s disease. *J. Parkinsons Dis.* 3 461–491. 10.3233/JPD-130230 24252804 PMC4135313

[B25] Dorman D. C. Struve M. F. Wong B. A. Dye J. A. Robertson I. D. (2006). Correlation of brain magnetic resonance imaging changes with pallidal manganese concentrations in rhesus monkeys following subchronic manganese inhalation. *Toxicol. Sci.* 92 219–227. 10.1093/toxsci/kfj209 16638924

[B26] El-Dawy K. Mohamed D. Abdou Z. (2022). Nanoformulations of pentacyclic triterpenoids: Chemoprevention and anticancer. *Int. J. Vet. Sci.* 11 384–391. 10.47278/journal.ijvs/2021.100

[B27] Elghobashy K. A. Eldanasoury M. M. Elhadary A. A. Farid M. (2020). Phytochemical constituent, HPLC profiling and antioxidant activity of *Passiflora incarnata* and *Arctium lappa* leaves extracts. *Int. J. Vet. Sci.* 9 42–49.

[B28] Elmore S. (2007). Apoptosis: A review of programmed cell death. *Toxicol. Pathol.* 35 495–516. 10.1080/01926230701320337 17562483 PMC2117903

[B29] Ema M. Hougaard K. S. Kishimoto A. Honda K. (2016). Reproductive and developmental toxicity of carbon-based nanomaterials: A literature review. *Nanotoxicology* 10 391–412. 26375634 10.3109/17435390.2015.1073811

[B30] Feng X. L. Chen A. J. Zhang Y. L. Wang J. F. Shao L. Q. Wei L. M. (2015). Central nervous system toxicity of metallic nanoparticles. *Int. J. Nanomed.* 10 4321–4340. 10.2147/IJN.S78308 26170667 PMC4498719

[B31] Filon F. L. Mauro M. Adami G. Bovenzi M. Crosera M. (2015). Nanoparticles skin absorption: New aspects for a safety profile evaluation. *Regul. Toxicol. Pharmacol.* 72 310–322. 25979643 10.1016/j.yrtph.2015.05.005

[B32] Fröhlich E. Salar-Behzadi S. (2014). Toxicological assessment of inhaled nanoparticles: Role of in vivo, ex vivo, in vitro, and in silico studies. *Int. J. Mol. Sci.* 15 4795–4822. 10.3390/ijms15034795 24646916 PMC3975425

[B33] Gabriele Sandrian M. Wollstein G. Schuman J. S. Bilonick R. A. Ling Y. Ishikawa H. (2012). Inflammatory response to intravitreal injection of gold nanorods. *Br. J. Ophthalmol.* 96 1522–1529. 10.1136/bjophthalmol-2012-301904 23087415 PMC3718482

[B34] Gao L. Yang S. T. Li S. Meng Y. Wang H. Lei H. (2013). Acute toxicity of zinc oxide nanoparticles to the rat olfactory system after intranasal instillation. *J. Appl. Toxicol.* 33 1079–1088. 10.1002/jat.2842 23315988

[B35] Geiser M. Jeannet N. Fierz M. Burtscher H. (2017). Evaluating adverse effects of inhaled nanoparticles by realistic in vitro technology. *Nanomaterials* 7:49. 10.3390/nano7020049 28336883 PMC5333034

[B36] Godfrey L. Iannitelli A. Garrett N. L. Moger J. Imbert I. King T. (2018). Nanoparticulate peptide delivery exclusively to the brain produces tolerance free analgesia. *J. Control Release* 270 135–144. 10.1016/j.jconrel.2017.11.041 29191784

[B37] Gosens I. Cassee F. R. Zanella M. Manodori L. Brunelli A. Costa A. L. (2016). Organ burden and pulmonary toxicity of nano-sized copper (II) oxide particles after short-term inhalation exposure. *Nanotoxicology* 10 1084–1095. 10.3109/17435390.2016.1172678 27132941 PMC4975088

[B38] Grueber W. B. Yang C. H. Ye B. Jan Y. N. (2005). The development of neuronal morphology in insects. *Curr. Biol.* 15 R730–R738. 16139206 10.1016/j.cub.2005.08.023

[B39] Hanley C. Thurber A. Hanna C. Punnoose A. Zhang J. H. Wingett D. G. (2009). The influences of cell type and ZnO nanoparticle size on immune cell cytotoxicity and cytokine induction. *Nanoscale Res. Lett.* 4 1409–1420. 10.1007/s11671-009-9413-8 20652105 PMC2894345

[B40] Harris M. Fallot R. D. (2001). Envisioning a trauma-informed service system: A vital paradigm shift. *New Direct. Ment. Health Serv.* 89 3–22. 10.1002/yd.23320018903 11291260

[B41] He C. C. Wei Y. J. Sun K. Li B. Dong X. Zou Z. (2013). Beclin 2 functions in autophagy, degradation of G protein-coupled receptors, and metabolism. *Cell* 154 1085–1099. 10.1016/j.cell.2013.07.035 23954414 PMC4231430

[B42] Hemmink J. D. Morgan S. B. Aramouni M. Everett H. Salguero F. J. Canini L. (2016). Distinct immune responses and virus shedding in pigs following aerosol, intranasal and contact infection with pandemic swine infuenza A virus, A(H1N1)09. *Vet. Res.* 47:103. 10.1186/s13567-016-0390-5 27765064 PMC5073419

[B43] Hildeman D. A. (2004). Regulation of T-cell apoptosis by reactive oxygen species. *Free Radic. Biol. Med.* 36 1496–1504. 10.1016/j.freeradbiomed.2004.03.023 15182852

[B44] Hillyer J. F. Albrecht R. M. (2001). Gastrointestinal persorption and tissue distribution of differently sized colloidal gold nanoparticles. *J. Pharm. Sci.* 90 1927–1936. 10.1002/jps.1143 11745751

[B45] Jalil P. J. Shnawa B. H. Hamad S. M. (2021). Silver nanoparticles: Green synthesis, characterization, blood compatibility, and protoscolicidal efficacy against *Echinococcus granulosus*. *Pak. Vet. J.* 41 393–399. 10.29261/pakvetj/2021.039

[B46] Kandeel M. Rehman T. U. Akhtar T. Zaheer T. Ahmad S. Ashraf U. (2022). Anti parasitic applications of nanoparticles: A review. *Pak. Vet. J.* 42 135–140.

[B47] Kanter M. Unsal C. Aktas C. Erboga M. (2016). Neuroprotective effect of quercetin against oxidative damage and neuronal apoptosis caused by cadmium in hippocampus. *Toxicol. Ind. Health* 32 541–550. 10.1177/0748233713504810 24193051

[B48] Kenzaoui B. H. Bernasconi C. C. Guney-Ayra S. Juillerat-Jeanneret L. (2012). Induction of oxidative stress, lysosome activation and autophagy by nanoparticles in human brain-derived endothelial cells. *Biochem. J.* 441 813–821. 10.1042/BJ20111252 22026563

[B49] Khan I. Zaneb H. Masood S. Ashraf S. Rehman H. F. Rehman H. U. (2022). Supplemental selenium nanoparticles-loaded to chitosan improves meat quality, pectoral muscle histology, tibia bone morphometry and tissue mineral retention in broilers. *Pak. Vet. J.* 42 236–240.

[B50] Kim I. D. Sawicki E. Lee H. K. Lee E. H. Park H. J. Han P. L. (2016). Robust neuroprotective efects of intranasally delivered iNOS siRNA encapsulated in gelatin nanoparticles in the postischemic brain. *Nanomedicine* 12 1219–1229. 10.1016/j.nano.2016.01.002 26945975

[B51] Kofroñová M. Hrdinová A. Mašková P. Tremlová J. Soudek P. Petrová Š (2020). Multi-component antioxidative system and robust carbohydrate status, the essence of plant arsenic tolerance. *Antioxidants* 9:283. 10.3390/antiox9040283 32230748 PMC7222215

[B52] Kwon J. T. Seo G. B. Jo E. Lee M. Kim H. M. Shim I. (2013). Aluminum nanoparticles induce ERK and p38MAPK activation in rat brain. *Toxicol. Res.* 29 181–185. 10.5487/TR.2013.29.3.181 24386518 PMC3877997

[B53] Li J. O W. Li W. Jiang Z. G. Ghanbari H. A. (2013). Oxidative stress and neurodegenerative disorders. *Int. J. Mol. Sci.* 14 24438–24475. 10.3390/ijms141224438 24351827 PMC3876121

[B54] Li Q. L. Mahendra S. Lyon D. Y. Brunet L. Liga M. V. Li D. (2008). Antimicrobial nanomaterials for water disinfection and microbial control: Potential applications and implications. *Water Res.* 42 4591–4602. 10.1016/j.watres.2008.08.015 18804836

[B55] Li Y. Wang C. Zong S. Qi J. Dong X. Zhao W. (2019). The trigeminal pathway dominates the nose-to-brain transportation of intact polymeric nanoparticles: Evidence from aggregation-caused quenching probes. *J. Biomed. Nanotechnol.* 15 686–702. 10.1166/jbn.2019.2724 30841963

[B56] Liang H. Chen A. Lai X. Liu J. Wu J. Kang Y. (2018). Neuroinfammation is induced by tongue-instilled ZnO nanoparticles via the Ca2+-dependent NF-kappaB and MAPK pathways. *Part Fibre Toxicol.* 15:39. 10.1186/s12989-018-0274-0 30340606 PMC6194560

[B57] Liu H. Ma L. Zhao J. Liu J. Yan J. Ruan J. (2009). Biochemical toxicity of nano-anatase TiO2 particles in mice. *Biol. Trace Element Res.* 129 170–180. 10.1007/s12011-008-8285-6 19066734

[B58] Liu S. C. Xu L. J. Zhang T. Ren G. G. Yang Z. (2010). Oxidative stress and apoptosis induced by nanosized titanium dioxide in PC12 cells. *Toxicology* 267 172–177. 10.1016/j.tox.2009.11.012 19922763

[B59] Liu Y. Gao Y. Liu Y. Li B. Chen C. Wu G. (2014). Oxidative stress and acute changes in murine brain tissues after nasal instillation of copper particles with diferent sizes. *J. Nanosci. Nanotechnol.* 14 4534–4540. 10.1166/jnn.2014.8290 24738425

[B60] Lopes V. R. Loitto V. Audinot J. N. Bayat N. Gutleb A. C. Cristobal S. (2016). Dose-dependent autophagic effect of titanium dioxide nanoparticles in human HaCaT cells at non-cytotoxic levels. *J. Nanobiotechnol.* 14:22. 10.1186/s12951-016-0174-0 27001369 PMC4802894

[B61] Lundquist P. Artursson P. (2016). Oral absorption of peptides and nanoparticles across the human intestine: Opportunities, limitations and studies in human tissues. *Adv. Drug Deliv. Rev.* 106 256–276. 10.1016/j.addr.2016.07.007 27496705

[B62] Maiuri M. C. Zalckvar E. Kimchi A. Kroemer G. (2007). Self-eating and self-killing: Crosstalk between autophagy and apoptosis. *Nat. Rev. Mol. Cell Biol.* 8 741–752. 10.1038/nrm2239 17717517

[B63] Marano F. Hussain S. Rodrigues-Lima F. Baeza-Squiban A. Boland S. (2011). Nanoparticles: Molecular targets and cell signalling. *Arch. Toxicol.* 85 733–741. 10.1007/s00204-010-0546-4 20502881

[B64] Massaad C. A. Klann E. (2011). Reactive oxygen species in the regulation of synaptic plasticity and memory. *Antioxid. Redox Signal.* 14 2013–2054. 10.1089/ars.2010.3208 20649473 PMC3078504

[B65] Mauro M. (2018). Nanoparticles skin exposure and absorption: Differences between children and adults. *Adv. Clin. Toxicol.* 3:000132. 10.23880/ACT-16000132 25139317

[B66] Mizushima N. Komatsu M. (2011). Autophagy: Renovation of cells and tissues. *Cell* 147 728–741. 10.1016/j.cell.2011.10.026 22078875

[B67] Momin J. K. Jayakumar C. Prajapati J. B. (2013). Potential of nanotechnology in functional foods. *Emir. J. Food Agric.* 25 10–19. 10.9755/ejfa.v25i1.9368

[B68] Moseman E. A. Blanchard A. C. Nayak D. McGavern D. B. (2020). T cell engagement of cross-presenting microglia protects the brain from a nasal virus infection. *Sci. Immunol.* 5:eabb1817. 10.1126/sciimmunol.abb1817 PMC7416530 32503876

[B69] Nel A. E. Madler L. Velegol D. Xia T. Hoek E. M. Somasundaran P. (2009). Understanding biophysico-chemical interactions at the nano-bio interface. *Nat. Mater.* 8 543–557. 10.1038/nmat2442 19525947

[B70] Nel A. Xia T. Madler L. Li N. (2006). Toxic potential of materials at the nanolevel. *Science* 311 622–627. 10.1126/science.1114397 16456071

[B71] Palmer B. C. DeLouise L. A. (2016). Nanoparticle-enabled transdermal drug delivery systems for enhanced dose control and tissue targeting. *Molecules.* 21:1719. 10.3390/molecules21121719 27983701 PMC5639878

[B72] Park H. A. Broman K. Stumpf A. Kazyak S. Jonas E. A. (2018). Nutritional regulators of Bcl-xL in the brain. *Molecules* 23:3019. 10.3390/molecules23113019 30463183 PMC6278276

[B73] Park J. S. Davis R. L. Sue C. M. (2018). Mitochondrial dysfunction in Parkinson’s disease: New mechanistic insights and therapeutic perspectives. *Curr. Neurol. Neurosci. Rep.* 18 1–11. 10.1007/s11910-018-0829-3 29616350 PMC5882770

[B74] Poljak-Blaži M. Jaganjac M. Žarković N. (2010). *Cell oxidative stress: Risk of metal nanoparticles.* London; New York, NY: CRC Press Taylor.

[B75] Pujalté I. Passagne I. Brouillaud B. Tréguer M. Durand E. Ohayon-Courtès C. (2011). Cytotoxicity and oxidative stress induced by different metallic nanoparticles on human kidney cells. *Part. Fibre Toxicol.* 8 1–16. 10.1186/1743-8977-8-10 21371295 PMC3058043

[B76] Qin X. Zhang J. Wang B. Xu G. Zou Z. (2017). LAMP-2 mediates oxidative stress-dependent cell death in Zn^2+^-treated lung epithelium cells. *Biochem. Biophys. Res. Commun.* 488 177–181. 10.1016/j.bbrc.2017.05.030 28483530

[B77] Rang F. J. Boonstra J. (2014). Causes and consequences of age-related changes in DNA methylation: A role for ROS? *Biology* 3 403–425. 10.3390/biology3020403 24945102 PMC4085615

[B78] Samy A. Hassan H. M. A. Elsherif H. M. R. (2022). Effect of nano zinc oxide and traditional zinc (oxide and sulphate) sources on performance, bone characteristics and physiological parameters of broiler chicks. *Int. J. Vet. Sci.* 11 486–492. 10.47278/journal.ijvs/2022.129 36500652 PMC9738599

[B79] Sánchez-Rodríguez E. Moreno D. A. Ferreres F. del Mar Rubio-Wilhelmi M. Ruiz J. M. (2011). Differential responses of five cherry tomato varieties to water stress: Changes on phenolic metabolites and related enzymes. *Phytochemistry* 72 723–729. 10.1016/j.phytochem.2011.02.011 21420135

[B80] Sarwar I. Ashar A. Mahfooz A. Aqib A. I. Saleem M. I. Butt A. A. (2021). Evaluation of antibacterial potential of raw turmeric, nano-turmeric, and NSAIDs against multiple drug resistant *Staphylococcus aureus* and *E. coli* isolated from animal wounds. *Pak. Vet. J.* 41 209–214.

[B81] Sharma A. Liaw K. Sharma R. Zhang Z. Kannan S. Kannan R. M. (2018). Targeting mitochondrial dysfunction and oxidative stress in activated microglia using dendrimer-based therapeutics. *Theranostics* 8 5529–5547. 10.7150/thno.29039 30555562 PMC6276292

[B82] Singh R. Nalwa H. S. (2011). Medical applications of nanoparticles in biological imaging, cell labeling, antimicrobial agents, and anticancer nanodrugs. *J. Biomed. Nanotechnol.* 7 489–503. 10.1166/jbn.2011.1324 21870454

[B83] Smith J. A. Park S. Krause J. S. Banik N. L. (2013). Oxidative stress, DNA damage, and the telomeric complex as therapeutic targets in acute neurodegeneration. *Neurochem. Int.* 62 764–775. 10.1016/j.neuint.2013.02.013 23422879 PMC3619128

[B84] Sohal I. S. O’Fallon K. S. Gaines P. Demokritou P. Bello D. (2018). Ingested engineered nanomaterials: State of science in nanotoxicity testing and future research needs. *Part. Fib. Toxicol.* 15 1–31. 10.1186/s12989-018-0265-1 PMC6029122 29970114

[B85] Sorce S. Krause K. H. (2009). NOX enzymes in the central nervous system: From signaling to disease. *Antioxid. Redox Signal.* 11 2481–2504. 10.1089/ars.2009.2578 19309263

[B86] Spruston N. Johnston D. (2008). Out of control in the dendrites. *Nat. Neurosci.* 11 733–734. 18575467 10.1038/nn0708-733

[B87] Sufian M. M. Khattak J. Z. K. Yousaf S. Rana M. S. (2017). Safety issues associated with the use of nanoparticles in human body. *Photodiagn. Photodyn. Ther.* 19 67–72. 10.1016/j.pdpdt.2017.05.012 28552731

[B88] Tammam A. M. Ibrahim S. A. Hemid A. A. Abdel-Azeem F. Salem W. (2020). Effect of nanoparticles supplementation in broiler diets on performance, microbial population and digestive tract measurements. *Int. J. Vet. Sci.* 9 373–378. 22889095

[B89] Tang J. Xiong L. Wang S. Wang J. Liu L. Li J. (2009). Distribution, translocation and accumulation of silver nanoparticles in rats. *J. Nanosci. Nanotechnol.* 9 4924–4932. 10.1166/jnn.2009.1269 19928170

[B90] Tapeinos C. Battaglini M. Ciofani G. (2017). Advances in the design of solid lipid nanoparticles and nanostructured lipid carriers for targeting brain diseases. *J. Contr. Rel.* 264 306–332. 10.1016/j.jconrel.2017.08.033 PMC6701993 28844756

[B91] Teixeira M. Sanchez-Lopez E. Espina M. Calpena A. Silva A. Veiga F. (2018). *Emerging nanotechnologies in immunology.* Amsterdam: Elsevier.

[B92] Torres M. Forman H. J. (2003). Redox signaling and the map kinase pathways. *BioFactors* 17 287–296. 10.1002/biof.5520170128 12897450

[B93] van der Zande M. Vandebriel R. J. Van Doren E. Kramer E. Rivera Z. H. Serrano-Rojero C. S. (2012). Distribution, elimination, and toxicity of silver nanoparticles and silver ions in rats after 28-day oral exposure. *ACS Nano* 6 7427–7442. 10.1021/nn302649p 22857815

[B94] von Bohlen und Halbach O. (2007). Immunohistological markers for staging neurogenesis in adult hippocampus. *Cell Tissue Res.* 329 409–420. 10.1007/s00441-007-0432-4 17541643

[B95] Warheit D. Sayes C. (2015). *Nanoengineering*, ed. Dolez P. I. (Amsterdam: Elsevier), 42.

[B96] Wiechers J. W. Musee N. (2010). Engineered inorganic nanoparticles and cosmetics: Facts, issues, knowledge gaps and challenges. *J. Biomed. Nanotechnol.* 6 408–431. 10.1166/jbn.2010.1143 21329039

[B97] Wirawan E. Vande Walle L. Kersse K. Cornelis S. Claerhout S. Vanoverberghe I. (2010). Caspase-mediated cleavage of Beclin-1 inactivates Beclin-1-induced autophagy and enhances apoptosis by promoting the release of proapoptotic factors from mitochondria. *Cell Death Dis.* 1:e18. 10.1038/cddis.2009.16 21364619 PMC3032505

[B98] Wu J. Ding T. Sun J. (2013). Neurotoxic potential of iron oxide nanoparticles in the rat brain striatum and hippocampus. *Neurotoxicology* 34 243–253. 22995439 10.1016/j.neuro.2012.09.006

[B99] Yang C. H. Huang Y. C. Tsai M. L. Cheng C. Y. Liu L. L. Yen Y. W. (2015). Inhibition of melanogenesis by beta-caryophyllene from lime mint essential oil in mouse B16 melanoma cells. *Int. J. Cosmet. Sci.* 37 550–554. 10.1111/ics.12224 25819153

[B100] Youssef F. S. Elbanna H. A. Elzorba H. Y. Galal A. M. Mohamed G. G. Ismail S. H. (2020). Synthesis and characterization of florfenicol-silver nanocomposite and its antibacterial activity against some gram positive and gram-negative bacteria. *Int. J. Vet. Sci.* 9 324–330.

[B101] Zaheer T. Ali M. M. Abbas R. Z. Atta K. Amjad I. Suleman A. (2022). Insights into nanopesticides for ticks: The superbugs of livestock. *Oxid. Med. Cell. Longev.* 18:7411481. 10.1155/2022/7411481 35720185 PMC9200545

[B102] Ze Y. Hu R. Wang X. Sang X. Ze X. Li B. (2014). Neurotoxicity and gene-expressed profle in brain-injured mice caused by exposure to titanium dioxide nanoparticles. *J. Biomed. Mater. Res. A* 102 470–478. 10.1002/jbm.a.34705 23533084

[B103] Zhang J. Wu L. Chan H.-K. Watanabe W. (2011). Formation, characterization, and fate of inhaled drug nanoparticles. *Adv. Drug Deliv.Rev.* 63 441–455. 10.1016/j.addr.2010.11.002 21118707

[B104] Zhang Y. Zhang W. Lobler M. Schmitz K. P. Saulnier P. Perrier T. (2011). Inner ear biocompatibility of lipid nanocapsules after round window membrane application. *Int. J. Pharm.* 404 211–219. 10.1016/j.ijpharm.2010.11.006 21075187

